# Optimizing Photoelectrochemical UV Imaging Photodetection: Construction of Anatase/Rutile Heterophase Homojunctions and Oxygen Vacancies Engineering in MOF-Derived TiO_2_

**DOI:** 10.3390/molecules29133096

**Published:** 2024-06-28

**Authors:** Yueying Ma, Yuewu Huang, Ju Huang, Zewu Xu, Yanbin Yang, Changmiao Xie, Bingke Zhang, Guanghong Ao, Zhendong Fu, Aimin Li, Dongbo Wang, Liancheng Zhao

**Affiliations:** 1 School of Science, Harbin University of Science and Technology, Harbin 150080, China; 2 School of Materials Science and Chemical Engineering, Harbin University of Science and Technology, Harbin 150080, China; 3 Department of Opto-Electronic Information Science, School of Materials Science and Engineering, Harbin Institute of Technology, Harbin 150080, China; 4 Tianjin Jinhang Technical Physics Institute, Tianjin 300308, China

**Keywords:** UV photodetector, self-powered, TiO_2_, heterophase homojunctions, UV imaging

## Abstract

Self-powered photoelectrochemical (PEC) ultraviolet photodetectors (UVPDs) are promising for next-generation energy-saving and highly integrated optoelectronic systems. Constructing a heterojunction is an effective strategy to increase the photodetection performance of PEC UVPDs because it can promote the separation and transfer of photogenerated carriers. However, both crystal defects and lattice mismatch lead to deteriorated device performance. Here, we introduce a structural regulation strategy to prepare TiO_2_ anatase-rutile heterophase homojunctions (A-R HHs) with oxygen vacancies (OVs) photoanodes through an in situ topological transformation of titanium metal–organic framework (Ti-MOF) by pyrolysis treatment. The cooperative interaction between A-R HHs and OVs suppresses carrier recombination and accelerates carrier transport, thereby significantly enhancing the photodetection performance of PEC UVPDs. The obtained device realizes a high on/off ratio of 10,752, a remarkable responsivity of 24.15 mA W^−1^, an impressive detectivity of 3.28 × 10^11^ Jones, and excellent cycling stability. More importantly, under 365 nm light illumination, a high-resolution image of “HUST” (the abbreviation of Harbin University of Science and Technology) was obtained perfectly, confirming the excellent optical imaging capability of the device. This research not only presents an advanced methodology for constructing TiO_2_-based PEC UVPDs, but also provides strategic guidance for enhancing their performance and practical applications.

## 1. Introduction

Ultraviolet photodetectors (UVPDs) capable of converting perceived light signals into electrical ones have attracted widespread attention with applications in photocontrol switches, environmental monitoring, biological analysis, and optical sensing imaging [1,2]. Among them, photoelectrochemical (PEC) UVPDs not only exhibit outstanding sensitivity but also circumvent the complex photolithography process associated with the fabrication of UVPDs based on the p-n junction or Schottky junction. Moreover, PEC UVPDs are capable of attaining a self-powered operation mode using the interfacial electric field between semiconductors and electrolytes, rendering them an ideal selection for wireless integrated energy-saving optoelectronic systems. Recently, TiO_2_ has been extensively employed as a photosensitive material in self-powered PEC UVPDs due to its facile nanostructure modification, low cost, security, and environmental friendliness [3]. However, current PEC UVPDs relying on pure TiO_2_ exhibit low light-harvesting capabilities, inadequate separation and transportation of photogenerated carriers, and interfacial charge recombination losses, which impact their performance and stability. As such, there is an urgent need for innovative TiO_2_ photoanode architectures that can suppress interfacial charge recombination while facilitating carrier transport, aiming to enhance the practical applications of PEC UVPDs. 

Optimizing the nanostructure of TiO_2_ photoanodes by morphology, porous modification, and hierarchical architecture offers advantages such as improving the specific surface area of the nanostructures and inducing multiple refractions of light, making them suitable for enhancing light-harvesting efficiency [4,5,6,7]. However, the agglomeration of the nanostructure impedes their development, as such strategies increase the charge transfer pathway and lead to obstruction of charge transport within the materials. Coating TiO_2_ with conductive networks of graphene, MXenes, and polymers is ideal for PEC UVPDs. These networks act as a scaffold, facilitating charge transport in the TiO_2_ films [8]. However, the poor attachment between the TiO_2_ nanostructures and conductive networks makes them susceptible to increased charge transfer resistance when exposed to electrolyte environments. Furthermore, the high density of surface states within conductive networks leads to nonradiative carrier recombination, posing challenges to further enhancing the photodetection performance of the devices [9]. Recently, researchers have attempted to couple different semiconductors to form heterojunctions with energy barriers and interfacial effects to enhance light harvesting and charge separation [10]. However, the passivation of effective nucleation during heterojunction growth leads to an increase in crystal defects, increasing the dark current of PEC UVPDs. Moreover, the lattice stress and strain induced by the lattice mismatch at the junction could impair the photodetection performance of the devices [11]. In this regard, leveraging the diverse crystal structures of TiO_2_ to develop anatase-rutile (A-R) heterophase homojunctions (HHs) holds promise as a strategy to overcome these issues [12,13,14]. The literature has reported that oxygen vacancies (OVs) can act as electron donors to promote the transfer of photogenerated carriers, which increases the conductivity and charge transport of TiO_2_, thus enhancing the photodetection performance of devices [15]. Therefore, the simple and rational preparation of TiO_2_ A-R HHs with OVs and their application in photoanodes are of great significance for engineering high-performance PEC UVPDs.

Here, we propose a strategy to utilize the in situ topological conversion process of titanium metal–organic frameworks (Ti-MOFs) for preparing MOF-derived TiO_2_ A-R HHs enriched with OVs and enhancing the photodetection performance of TiO_2_-based PEC UVPDs. The moderate OVs and the Type II band alignment of A-R HHs contribute to the enhanced separation of photogenerated electron-hole pairs in TiO_2_. More importantly, we verify that the presence of a barrier layer in the built-in electric field of TiO_2_ A-R HHs effectively reduces the recombination loss of photogenerated carriers with I_3_^−^ in the Helmholtz layer. Benefiting from the combined impact of improving the separation of photogenerated electron-hole pairs and reducing carrier recombination, the resulting device achieves a high on/off ratio of 10,752, a remarkable responsivity of 24.15 mA W^−1^, an impressive detectivity of 3.28 × 10^11^ Jones, and excellent cycling stability under 365 nm irradiation at 0 V bias. Validating the application, the device integrated into the optical system as the sensing pixel achieves excellent high-resolution imaging, providing robust support for the future development of high-performance, multifunctional optoelectronic systems.

## 2. Results and Discussion

**Figure 1:**
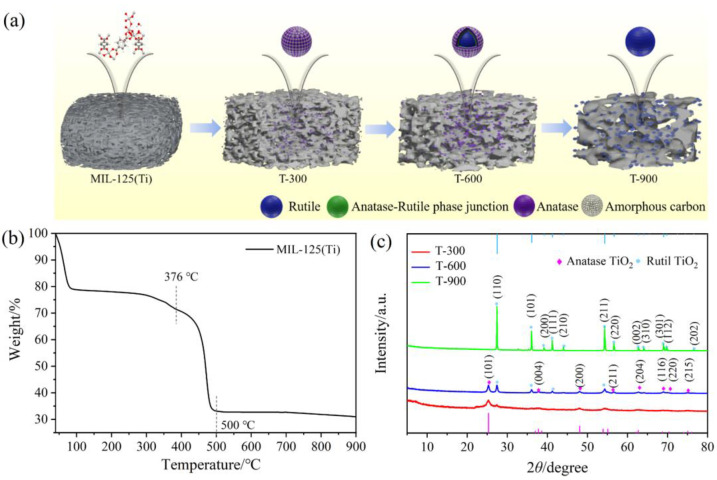
(**a**) Illustration of the in situ topological conversion process of MIL-125(Ti). (**b**) TG curve of the as-synthesized MIL-125(Ti). (**c**) XRD patterns of T-300, T-600, and T-900.

The plausible growth procedure, featuring an in situ topological conversion strategy for MOF-derived TiO_2_ A-R HHs, is illustrated in Figure 1a. During the initial stage, mooncake-like MIL-125(Ti) was fabricated and employed as templates. Then, the formed Ti-MOF decomposed into TiO_2_ A-R HHs, while the organic linkers created a porous carbon matrix during pyrolysis treatment [16]. The crystallographic structure of the as-synthesized MIL-125(Ti) was examined by XRD analysis. As shown in Figure S1, the distinct peaks of the as-synthesized MIL-125(Ti) match well with the simulated XRD patterns, indicating the formation of pure phase MIL-125(Ti). To better understand the transition of MIL-125(Ti) into TiO_2_ as the temperature increases, TG analysis was performed up to 900 °C, as illustrated in Figure 1b. The initial weight loss of approximately 21% up to 100 °C is attributed to the removal of adsorbed gases and the vaporization of solvents or water molecules from the pores of Ti-MOF [17]. A subsequent weight loss of 9.2% around 376 °C corresponds to the breaking of coordination bonds between the organic linker and the Ti oxo-cluster [18]. At around 550 °C, a substantial 66% mass loss occurs primarily due to the decomposition of the organic ligand along with the disintegration of the framework structure [19]. Notably, the prepared MIL-125(Ti) shows a slight weight loss between 550 and 900 °C, indicating that a small quantity of carbon from the organic linker is retained in the MOF-derived TiO_2_.

As shown in Figure 1c, the diffraction peaks of T-300 display only broad and weak characteristics of A-phase TiO_2_ (JCPDS No. 21-1272), which suggests the possible presence of a substantial layer of carbon on the TiO_2_ matrix surfaces or the formation of TiO_2_ nanoparticles (NPs) with relatively low crystallinity [20]. The favored formation of the A-phase at lower pyrolysis temperatures can be understood through its structural characteristics. The unit cell of the A-phase, known for its long-range order, experiences minimal molecular restriction during the nucleation process, promoting its formation at temperatures lower than that required for the R-phase [21]. From a thermodynamic perspective, the lower temperature formation of the A-phase can be attributed to the differences in the Gibbs free energy. During nucleation, the A-phase exhibits a smaller change in surface-free energy than the R-phase of TiO_2_, indicating that it requires less energy from the surroundings to establish a stable nucleation surface. Therefore, the A-phase is more likely to form at lower temperatures compared to the R-phase [22]. With an increase in the pyrolysis temperature to 600 °C, the observed peaks at 2*θ* of 25.28°, 37.8°, 48.05°, 55.06°, 62.69°, 68.76°, 70.31°, and 75.03° correspond to the (101), (004), (200), (211), (204), (116), (220), and (215) crystal planes of A-phase TiO_2_ (JCPDS No. 21-1272), respectively. Furthermore, the diffraction peaks around 27.4°, 36.08°, 41.23°, and 54.33° align with those of R-phase TiO_2_ (JCPDS No. 21-1276), confirming the successful preparation of TiO_2_ A-R HHs in the T-600 sample. For the T-900 sample, the diffraction peaks of the R-phase were notably enhanced, while those associated with the A-phase were no longer detectable, confirming the transformation of MOF-derived TiO_2_ from a low-crystallinity metastable A-phase to a crystalline R-phase. Moreover, no additional peaks corresponding to carbon (JCPDS No. 75-0444) were detected in the T-600 and T-900 samples, indicating the formation of strong bonds between carbon and TiO_2_ during the pyrolysis process.

The A-phase to R-phase ratio (*W*_A_/*W*_R_, %) values for the obtained samples can be determined by calculating the peak intensity using the following formula [23] (as listed in Table 1):

(1) $$W_{A}=\frac{K_{A}I_{A}}{K_{A}I_{A}+I_{R}}$$

(2) $$W_{R}=1-W_{A}$$

where *K*_A_ is a coefficient with a value of 0.886, and *W*_A_ and *W*_R_ represent the relative contents of A-phase and R-phase, respectively. *I*_A_ and *I*_R_ correspond to the A-phase (110) and R-phase (101) diffraction peak areas, respectively. This reveals that an increase in pyrolysis temperature leads to a gradual increase in the content of the R-phase, which is likely attributable to the increase in the surface energy of TiO_2_ NPs at high temperature [24], further confirming the transformation from the A-phase to the R-phase. The elevated surface energy is conducive to the nucleation of the R-phase at the A-phase grain boundaries, promoting the formation of in situ TiO_2_ A-R HHs. In addition, sample T-600 displays an almost 1:1 A/R phase ratio, which is conducive to the formation of a phase junction.

The average crystalline sizes of T-300, T-600, and T-900 were determined using the Debye–Scherrer formula [25]:

(3) $$D=\frac{0.89\lambda }{\beta \cos\theta }$$

where *D* represents the average crystalline size, *λ* is 1.5406 Å, *β* is the full width at half maximum, and *θ* is the diffraction angle. The calculated *D* values of the A-phase and R-phase in the T-300, T-600, and T-900 samples are listed in Table 1. It can be observed that the D value for T-300 is significantly smaller than that of the other two samples, which could be a result of the inhibitory effect of carbon from the organic linker on the aggregation of smaller NPs [26]. Consequently, the pyrolysis treatment can be regarded as a feasible method for forming TiO_2_ A-R HHs.

N_2_ adsorption–desorption isotherms were employed to evaluate the influence of pyrolysis temperature on the textural properties of the as-synthesized samples. As shown in Figure S2 and Table 1, the MIL-125(Ti) possesses a specific surface area of 921.1 m^2^ g^−1^, exhibiting a characteristic microporous structure. Following annealing, both the T-300 and T-600 demonstrate clear H4 hysteresis loops at P/P_0_ > 0.6, signifying a transition from microporous to mesoporous structures. However, the specific surface area of T-600 decreases significantly from 246.7 to 23.7 m^2^ g^−1^, with an increase in the pore size from 3.6 to 20.5 nm compared to T-300. This phenomenon can be attributed to the incomplete conversion of MIL-125(Ti) into TiO_2_ at 300 °C, preserving a significant proportion of the Ti-MOF structure. With further increases in pyrolysis temperature, the Ti-MOF structure collapses and leads to the disappearance of numerous micropores and the formation of new mesopores during the re-stacking process, which agrees with the TG analysis results. Furthermore, the T-900 sample displays an N_2_ adsorption–desorption isotherm profile similar to that of MIL-125(Ti), particularly in its microporous architecture. However, the specific surface area of T-900 decreases significantly to 1.4 m^2^ g^−1^, which can be explained by the expansion of crystalline grain size associated with an increase in pyrolysis temperature.

**Figure 2:**
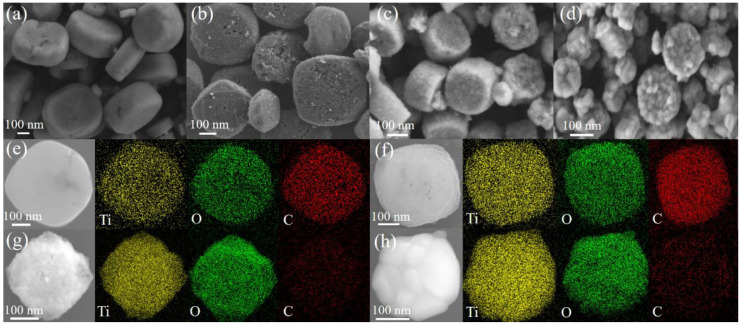
FESEM images of (**a**) MIL-125(Ti), (**b**) T-300, (**c**) T-600, and (**d**) T-900. FESEM and the corresponding elemental maps of (**e**) MIL-125(Ti), (**f**) T-300, (**g**) T-600, and (**h**) T-900.

The FESEM images reveal that the MIL-125(Ti) exhibits a consistent mooncake-like nanostructure, with lengths ranging from 140 to 500 nm and thicknesses ranging from 120 to 260 nm (Figure 2a and Figure S3). During pyrolysis, the coordination bonds between Ti oxo-clusters and organic linkers break, transforming Ti oxo-clusters into TiO_2_ NPs and the organic linkers into a surface-functionalized porous carbon matrix. This transformation preserves the mooncake-like structures, as displayed in Figure 2b–d. Furthermore, the MOF-derived TiO_2_ experiences a significant size reduction compared to MIL-125(Ti) due to the loss of carbon from the organic ligands, which is consistent with the TG analysis results. Figure 2e–h displays the corresponding EDS mappings of the as-synthesized samples, which reveals the uniform distribution of C, Ti, and O atoms across the samples, indicating the successful synthesis of TiO_2_ embedded in a continuous conductive carbon network via an in situ pyrolysis strategy. Notably, the weight percentage of carbon decreases with increasing temperature (Table 1 and Figure S4), even after the complete disintegration of the Ti-MOF structure (Figure 1b).

**Figure 3:**
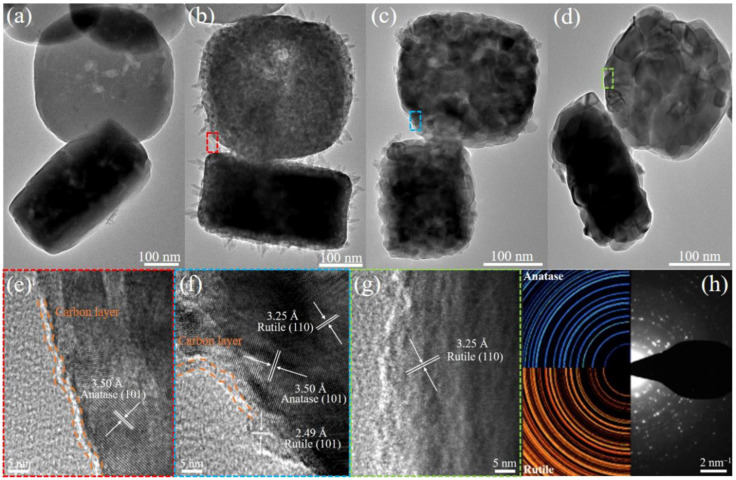
TEM images of (**a**) MIL-125(Ti), (**b**) T-300, (**c**) T-600, and (**d**) T-900. HRTEM images of (**e**) T-300, (**f**) T-600 and (**g**) T-900 (the corresponding locations are the areas marked by red, blue, and green squares in subfigure (**b**–**d**)). (**h**) SAED pattern of T-600.

The morphological and structural transformation of the as-synthesized samples were further characterized using TEM and HRTEM. As shown in Figure 3a–d, significant changes in the mooncake-like structure of the as-synthesized samples are observed with increasing pyrolysis temperature. For Ti-MOF (Figure 3a), the MIL-125(Ti) maintains a densely smooth mooncake-like structure. When the pyrolysis temperature reaches 300 °C (Figure 3b), the T-300 sample still maintains a mooncake-like structure, but its size significantly decreases compared to MIL-125(Ti). In addition, the surface of the T-300 sample changes from smooth to rough, composed of numerous interconnected TiO_2_ NPs (~6.4 nm), which is attributed to the formation of channels induced by gas evolution during the pyrolysis process. After further increasing the pyrolysis temperature (600 °C), it is observed that these cakes (T-600) exhibit characteristic mesoporous structures and contain numerous NPs with an average diameter of ~15.4 nm. As the pyrolysis temperature increases to 900 °C, the average size of the NPs in T-900 increases to ~36.3 nm, and the dark regions between adjacent NPs indicate a decrease in pore size (Figure 3d). The HRTEM image of T-300 (Figure 3e) exhibits an interplanar distance of 3.50 Å, corresponding to the (101) plane spacing of A-phase TiO_2_. Moreover, the lattice fringes with interplanar distances of 3.25 Å and 2.49 Å, shown in Figure 3f,g, are attributed to the (110) and (101) planes of R-phase TiO_2_, respectively. Furthermore, the SAED (Figure 3h) reveals diffraction rings corresponding to both the A-phase and R-phase, confirming the successful preparation of TiO_2_ A-R HHs. In addition, an amorphous carbon layer (~2 nm) is observed at the edges of T-300 and T-600 samples, while no evidence of carbon layers is found in the T-900, indicating carbon loss due to the high-temperature pyrolysis of Ti-MOF. The presence of the carbon layers offers additional transport pathways for photogenerated carriers, ensuring not only efficient light absorption but also suppressing the recombination of electron–hole pairs [27].

**Figure 4:**
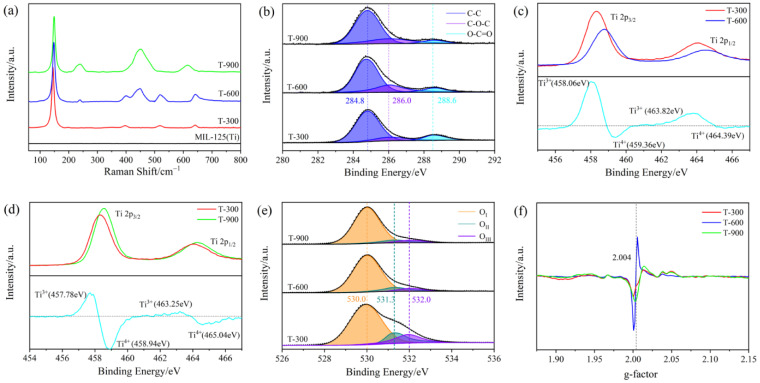
(**a**) Raman spectra. (**b**) C 1s XPS (**c**,**d**) Ti 2p XPS. (**e**) O 1s XPS and (**f**) EPR spectra of T-300, T-600, and T-900.

The MOF-derived TiO_2_ was analyzed using Raman spectroscopy to further understand its structural characteristics. As shown in Figure 4a, the characteristic Raman peaks of the A-phase identified at 145.0 cm^−1^ (E_g_), 397.0 cm^−1^ (B_1g_), 518.8 cm^−1^ (A_1g_), and 640.6 cm^−1^ (E_g_) are consistent with the previous literature [28]. Meanwhile, the peaks at 239.5, 449.4, and 614.5 cm^−1^ observed in T-600 and T-900 correspond to the B_1g_, E_g_, and A_1g_ modes of the R-phase, respectively [29]. With the increase in pyrolysis temperature, the magnified Raman peak in the 100–200 cm^−1^ range (Figure S5) shifts higher due to the disruption of Ti-O-Ti bonds, which affects the force constants and vibrational amplitudes of the nearest neighbor bonds, indicating the formation of OVs in the lattice of MOF-derived TiO_2_ [30]. Furthermore, the peak intensities at 239.5 and 449.4 cm^−1^ become more pronounced, and the peak at 518.8 cm^−1^ vanishes. The peak at 397.0 cm^−1^ migrates to higher frequencies, and the one at 640.6 cm^−1^ moves to lower frequencies, suggesting a phase transition from the metastable A-phase to the more stable R-phase with increasing pyrolysis temperature. The observations indicate that the T-600 sample simultaneously exhibits both the A-phase and R-phase, with a noticeable increase in the proportion of the R-phase as the temperature continues to rise, consistent with the earlier discussed XRD measurements.

XPS was used to analyze the chemical states and compositions of the MOF-derived TiO_2_ samples. The XPS survey spectra reveal the presence of C 1s, Ti 2p, and O 1s peaks in the as-synthesized samples (Figure S6). As shown in Figure 4b, the peaks observed at 284.8, 286.0, and 288.6 eV correspond to the C-C, C-O, and C=O bonds, respectively [31]. The C-C bond is related to the amount of amorphous carbon, while the C-O and C=O bonds are associated with the amount of organic carbonaceous species on the surface. The data obtained from quantitative analysis of the C chemical bonds are summarized in Table S1. Due to the high-temperature pyrolysis of the precursor, the percentages of C-O and C=O decrease in the T-600 and T-900, while the percentage of C-C gradually increases, which is consistent with the TG analysis results. By subtracting the normalized Ti 2p spectra of T-600 from that of T-300, two additional peaks at 458.06 and 463.82 eV are distinctly observed (Figure 4c). These peaks are attributed to the characteristic Ti 2p_3/2_ and Ti 2p_1/2_ peaks of Ti^3+^, confirming the generation of Ti^3+^ sites (OVs) during pyrolysis [32,33]. Similar results are observed in the Ti 2p XPS spectra of T-900 compared with T-300 (Figure 4d). More importantly, the binding energies of both Ti 2p_3/2_ and Ti 2p_1/2_ for T-900 are slightly lower than those of T-600, and the relative intensity of Ti^3+^ peaks for T-900 is notably high-er than that of T-600, revealing that more Ti^3+^ sites (OVs) were introduced and stabilized in T-900 compared to T-600. As shown in Figure 4e, the O 1s peaks can be resolved into three components centered at 530.0 (O_I_), 531.3 (O_II_), and 532.0 eV (O_III_), corresponding to the O^2−^ in the TiO_2_ lattice, the oxygen ions in oxygen-deficient regions, and OH-related species, respectively [34]. The ratio of peak area (O_II_/O_I_ + O_II_) is related to the relative content of OVs. As demonstrated in Table S1, the content of OVs in T-600 reduced from 16.2 to 9.9% compared to T-300, which can be attributed to the oxidation of surface Ti^3+^ to Ti^4+^ at a higher temperature [35]. Interestingly, the T-900 sample exhibited an unexpected increase in OVs to 11.4%, likely due to TiO_2_ with a high density of Ti^3+^ defects being exposed to an oxygen-rich environment. In such a setting, surface oxygen can easily compensate for the excess charge of Ti^3+^ and gradually fill the OVs, effectively transferring them from the surface to the bulk oxygen and leading to an increased formation of OVs in the TiO_2_ lattice at high temperatures. 

EPR, known for its exceptional sensitivity in detecting paramagnetic species such as unpaired electrons, was used to further confirm the presence of OVs. As shown in Figure 4f, all samples exhibit a notable signal peak at a g-value of 2.004, indicative of electrons trapped at OV sites, thereby substantiating the existence of OVs. It is worth noting that the EPR technique, not capable of directly detecting surface OVs, likely detects signals emanating from bulk single-electron-trapped oxygen vacancies (SETOVs) [36]. During the pyrolysis process, surface OVs tend to transform into bulk OVs, which explains why the EPR signal of T-600 is greater than that of T-300. Concurrently, the T-300 sample displays the lowest EPR signal due to the higher propensity of OVs to form in the A-phase compared to the R-phase, resulting in the creation of shallow donors. These findings are in agreement with the XPS results, which collectively provide evidence for the presence of OVs following the pyrolysis process.

**Figure 5:**
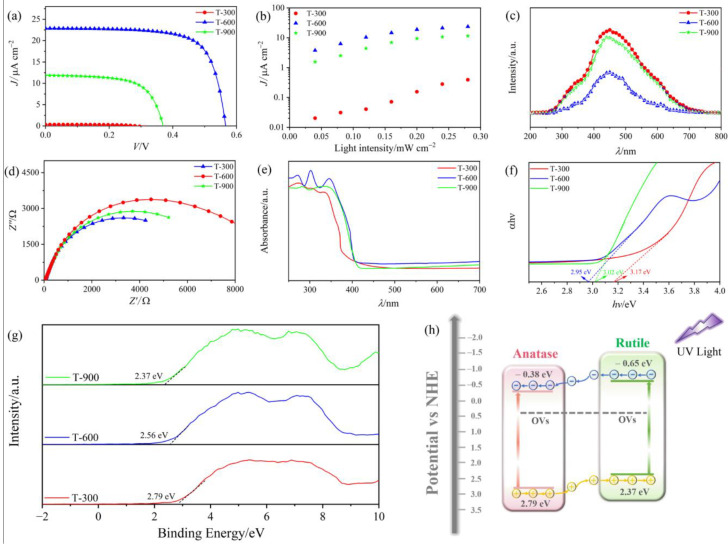
(**a**) *J*−*V* characteristics and (**b**) corresponding correlation between *J_sc_* and *P_λ_* of the PEC UVPDs with T-300, T-600, and T-900. (**c**) PL spectra, (**d**) Nyquist plots of EIS, (**e**) UV–Vis DRS, (**f**) Bandgap energy plots, and (**g**) VB-XPS spectra of T-300, T-600, and T-900. (**h**) Schematic illustration for the band structure of the TiO_2_ A-R HHs.

To elucidate the synergistic impact of A-R HHs and OVs on the performance of PEC UVPDs, their *J*−*V* characteristics were measured under 365 nm light irradiation with an intensity (*P_λ_*) of 0.24 mW cm^−2^, as illustrated in Figure 5a. It is found that as the pyrolysis temperature increases, the short-circuit current density (*J*_sc_) of the devices initially increases and then decreases. The peak value of *J*_sc_ is recorded as 22.65 μA cm^−2^ for the T-600 UVPD. To ensure the accuracy of the *J*_sc_ variation pattern, the *J*−*V* characteristic curves of the devices were tested under a range of *P_λ_*, varying from 0.04 to 0.28 mW cm^−2^. As shown in Figure 5b, the T-600 UVPD consistently exhibits the highest *J*_sc_, followed by T-900, and the lowest for T-300 at any given *P_λ_*. This consistent behavior indicates the excellent repeatability and reproducibility of the MOF-derived TiO_2_ PEC UVPDs. To gain deeper insight into the mechanism for the variations in the *J*_sc_ values, the PL spectra of MOF-derived TiO_2_ films were measured. As shown in Figure 5c, the PL spectra of the as-synthesized samples excited at 325 nm exhibit similar PL spectral profiles, with two broad emission bands observed in the 250–700 nm. The initial peak at approximately 450 nm is likely due to free exciton emission, while the subsequent peak around 465 nm is attributed to OVs [37]. Among all samples, the T-600 has the lowest PL intensity, suggesting that A-R HHs and OVs effectively suppress the recombination of photogenerated carriers. Moreover, the higher PL intensity in T-900 compared to T-600 can be attributed to an excess of OVs, which act as trapping centers that accelerate the recombination of photogenerated carriers and lead to reductions in *J*_sc_. To further elucidate the influence of pyrolysis temperature on charge separation and transport, the EIS measurements were conducted on the as-prepared samples in the dark. As shown in Figure 5d, the arcs observed in the EIS spectra are indicative of charge-transfer resistance (*R*_ct_) at the photoanode/electrolyte interface. Notably, the arc radius for T-600 exhibits the smallest value (*R*_ct_ = 6237.3 Ω) compared to T-300 and T-900, suggesting the highest separation and transfer efficiency of photogenerated carriers among the three samples.

To better understand the impact of varying pyrolysis temperatures on *J*_sc_, we further evaluated the charge carrier transport processes using M–S analysis. As shown in Figure S7, all samples exhibited positive slopes, indicating their inherent n-type semiconductor characteristics. Additionally, the charge carrier densities (*N*_d_) of the as-synthesized samples can be determined through the equation [38]:

(4) $$N_{d}=\left(\frac{2}{q\varepsilon \varepsilon_{0}}\right){\left[d\left(\frac{1}{C^{2}}\right)dV\right]}^{-1}$$

where *q* is the elementary charge, *ε* is the relative permittivity of TiO_2_ (*ε* = 48) [39], *ε*_0_ is the vacuum permittivity, and d(1/*C*^2^)/d*V* is the linear slope. The calculated *N*_d_ values of T-300, T-600, and T-900 are 0.63 × 10^16^, 0.41 × 10^17^, and 0.12 × 10^17^, respectively. In other words, the T-600 exhibits the highest *N*_d_ value, which is an order of magnitude greater than that observed in T-300 and T-900, contributing to the enhancement of *J*_sc_.

Figure 5e exhibits the UV-visible diffuse reflectance spectra of T-300, T-600, and T-900. Notably, the T-300 sample shows the lowest absorption edge, whereas a higher pyrolysis temperature leads to a shift of the absorption edge toward longer wavelengths. Tauc-plots, illustrated in Figure 5f, were utilized to determine the bandgaps of the three samples. The bandgaps for T-300 and T-900 are 3.17 eV and 3.02 eV, respectively, which is consistent with previously reported data [40]. Furthermore, the valence band (VB) positions for T-300 and T-900, as identified via VB–XPS and illustrated in Figure 5g, were determined to be 2.79 eV and 2.37 eV, respectively. Utilizing the equation *E*_CB_ = *E*_VB_ − *E*_g_, the conduction band (CB) of T-300 and T-900 were calculated to be −0.38 eV and −0.65 eV, respectively. The resultant electronic band structure diagram is illustrated in Figure 5h. The VB top and CB bottom of the R-phase is higher than those of the A-phase, suggesting the formation of type-II phase junctions between the R-phase and the A-phase. Consequently, a built-in electric field forms at the closely contacted interfaces, driving photogenerated electrons from the R-phase to the A-phase and photogenerated holes in the opposite direction, resulting in highly efficient separation. Moreover, the OVs serve as electron donors located 0.7–1.0 eV below the CB of TiO_2_, which increases the electric conductivity and thus promotes the separation and transfer of photogenerated carriers [41]. Based on the above discussions, the enhanced *J*_sc_ could be attributed to the synergistic effect between the enhancement of photogenerated carrier transfer characteristics by OVs acting as donors, and the effective inhibition of photogenerated carrier recombination by TiO_2_ A-R HHs.

**Figure 6:**
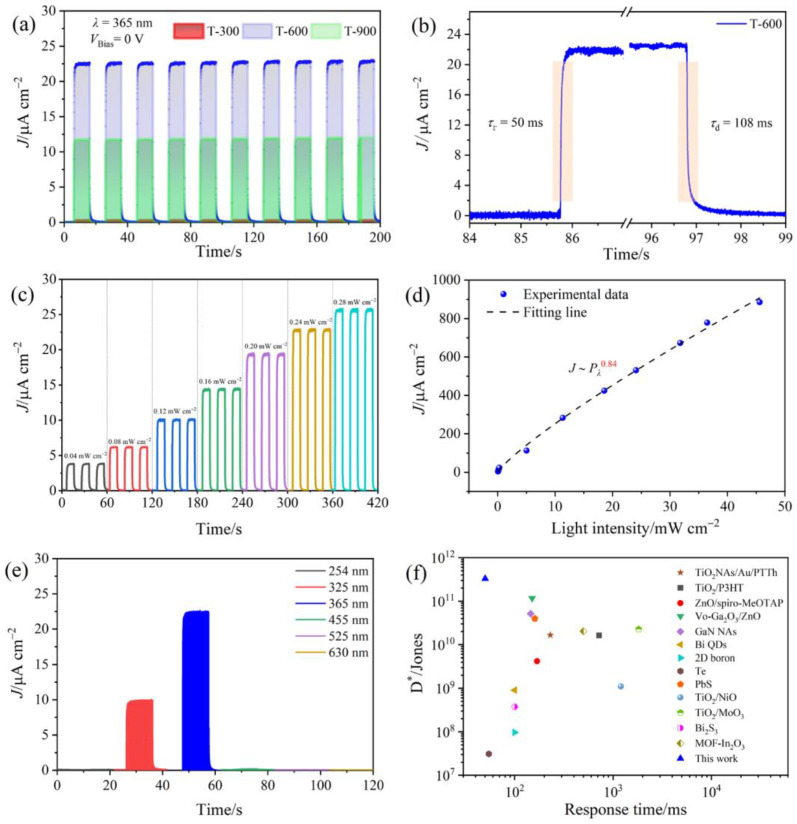
(**a**) Photocurrent responses of the PEC UVPDs with T-300, T-600, and T-900 under 0.24 mW cm^−2^ UV light illumination (*λ* = 365 nm). (**b**) Response speed of T-600 PEC UVPD. (**c**) Photocurrent response of T-600 PEC UVPD as a function of time under *P_λ_* from 0.04 to 0.28 mW cm^−2^. (**d**) *J* as a function of *P_λ_* from 0.04 to 45.61 mW cm^−2^ for the T-600 PEC UVPD. (**e**) Spectral photoresponse of the T-600 PEC UVPD irradiated by 254, 325, 365, 455, 525, and 630 nm. (**f**) Comparison of D* and response speed of nanomaterial-based self-powered UVPDs.

To further demonstrate the feasibility of using the prepared MOF-derived TiO_2_ as the photoanode material for PEC UVPDs, the photoresponse characteristics and switching behavior of the fabricated devices were examined under 0 V bias and UV light irradiation (*λ* = 365 nm, *P_λ_* = 0.24 mW cm^−2^). Figure 6a shows that all the devices exhibit stable and reproducible performance with a characteristic light-dependent response. The T-900 UVPD demonstrates an elevated on/off ratio of 5851 compared to the T-300 (159), indicating a notable improvement in photodetection performance due to the reduction of OVs. The T-600 UVPD further increases to 10,752 and highlights the advantageous impact of TiO_2_ A-R HHs. Response time serves as a crucial metric for assessing photodetection performance, indicating device sensitivity to UV light. Typically, the rise time (*τ*_r_) is defined as the duration required for the peak photocurrent density (*J*) to transition from 10% to 90%, while the decay time (*τ*_d_) is the period for *J* to decline from 90% to 10% of its maximum value. As shown in Figure 6b, the *τ*_r_ and *τ*_d_ of the T-600 UVPD at 0 V bias are 50 ms and 108 ms, respectively. Interestingly, the *τ*_r_/*τ*_d_ calculated for T-300 and T-900 are 276/51 ms and 149/73 ms, respectively (Figure S8). As a result, the T-600 UVPD possesses the fastest *τ*_r_, suggesting the highest transfer rate and separation efficiency of photogenerated carriers in T-600 among the three samples. However, the T-300 UVPD exhibits a relatively rapid *τ*_d_ compared to the other two devices, which is attributed to the heightened defect density resulting from subpar crystalline quality, leading to significant interfacial electron recombination. Figure 6c presents the time-dependent behavior of T-600 UVPD when exposed to 365 nm incident light across *P_λ_* ranging from 0.04 to 0.28 mW cm^−2^. Across three distinct cycles, there is a noticeable increase in the *J* value, correlating with the increase in *P_λ_*. This trend is likely since higher *P_λ_* induces the excitation of a greater number of photogenerated carriers, thereby yielding an amplified *J*. In addition, we also tested the time-dependent photocurrent of the T-600 UVPD under variable *P_λ_* ranging from 5.03 to 45.61 mW cm^−2^ (Figure S9). Figure 6d, derived from the data in Figure 6c and Figure S9, shows *J* as a function of *P_λ_*. It can be observed that *J* displays a power-law relationship, which can be modeled by the equation *J*~*P_λ_^α^*, where *α* is the power-law index [42]. The derived value of *α* is 0.84, suggesting that the T-600 device provides a more sensitive and accurate approach for the quantitative measurement of UV light.

Spectral selectivity is a key parameter in evaluating the performance of UVPDs. As shown in Figure 6e, the T-600 UVPD exhibits superior wavelength selectivity, with pronounced photoresponse to UVA (365 nm), decreased responsiveness to deep UV (254 nm), and negligible response to the broader visible spectrum. Figure S10 presents the trPL spectra of T-600 under 254 and 365 nm illumination. The average lifetime (*τ*_avg_) of photogenerated carriers, deduced from PL decay, is 0.14 ns for 254 nm and 1.5 ns for 365 nm. The shorter *τ*_avg_ at 254 nm implies accelerated carrier recombination, which explains the increasing trend of *J*. To assess the spectral photoresponse of the T-600 UVPD, its responsivity characteristic (*R*) was calculated using the following formula [43]:

(5) $$R=\frac{J-J_{d}}{P_{\lambda }}$$

where *J*_d_ is the dark current density. As shown in Figure S11, the responsivity peak of three devices is within the narrow wavelength range of 300–400 nm, precisely aligning with the UVA radiation band. In particular, the T-600 UVPD achieves a peak responsivity of 24.15 mA W^−1^ at 350 nm, indicating that the integration of A-R HHs and OVs can effectively boost the photoresponse performance of the device. In addition, the UV/visible light suppression ratio (R-350 nm/R-450 nm) of the T-600 device reaches 537, which significantly outperforms the T-300 device (R-350 nm/R-450 nm = 5) and the T-900 device (R-350 nm/R-450 nm = 54), demonstrating its excellent wavelength selectivity.

The detectivity (D*), a crucial metric for evaluating the sensitivity to faint light signals, was described by the equation [44]:

(6) $$D^{*}=\frac{R}{{\left(2qJ_{d}\right)}^{1/2}}$$

where *q* represents the elementary charge. At 0 V bias, the T-600 device exhibits a peak D* of 3.28 × 10^11^ Jones at 350 nm, which results from the reduction in OV concentration and the establishment of A-R HHs in the T-600 photoanode. To facilitate better comparison, Table S2 summarizes a comprehensive investigation of nanomaterial-based self-powered UVPDs with key parameters. As illustrated in Figure 6f, the device in this work exhibits a comparatively high D* value compared to the reported PEC UVPDs. Moreover, its overall performance even exceeds that of most self-powered UVPDs, which are not limited to the PEC type and are based on nanomaterial photoanodes [5,45,46,47,48,49,50,51,52,53,54,55,56]. All these merits indicate that this MOF-derived TiO_2_ with optimized OVs and A-R HHs has broad application prospects in high-performance UVPDs.

**Figure 7:**
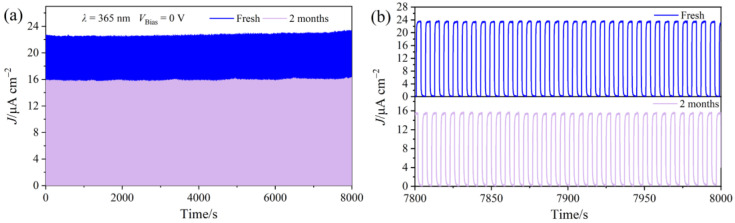
(**a**) Cycling stability assessments of fresh T-600 PEC UVPD and the same device stored in air for two months. (**b**) The detailed on/off signals within the 7800–8000 range in (**a**).

The stability of UVPDs is a critical factor in determining their future deployment potential. To evaluate the cyclic stability and long-term storage durability of the T-600 device, the instantaneous switching behavior was tested over 1300 cycles immediately after fabrication and again after two months of air storage. As shown in Figure 7a, the fresh device displays an initial *J* of 22.65 μA cm^−2^. After being stored under ambient conditions for two months without encapsulation, the T-600 device retains over 70% of its initial *J*, demonstrating superior stability and high tolerance. Figure 7b displays a magnified view of the initial time stability measurements, revealing that the *J* of the T-600 device exhibits excellent repeatability, with no significant attenuation observed under UV light irradiation (*λ* = 365 nm, *P_λ_* = 0.24 mW cm^−2^). More importantly, despite a decline of approximately 30% in *J* after two months of storage in air, the T-600 UVPD still demonstrates good repeatability. Furthermore, to assess the thermal stability of standard devices at 85 °C, an iodine-based electrolyte was not used due to its potential to impair detection performance. Instead, a low-volatility electrolyte (E1, more detail in Supplementary Materials) was used as a more suitable alternative for the thermal stability evaluation of the T-600 device. Figure S12 demonstrates that after 1000 hours of thermal aging at 85 °C, the device maintains over 94% of its initial *J*, substantiating the exceptional thermal stability of the T-600 UVPD. 

**Figure 8:**
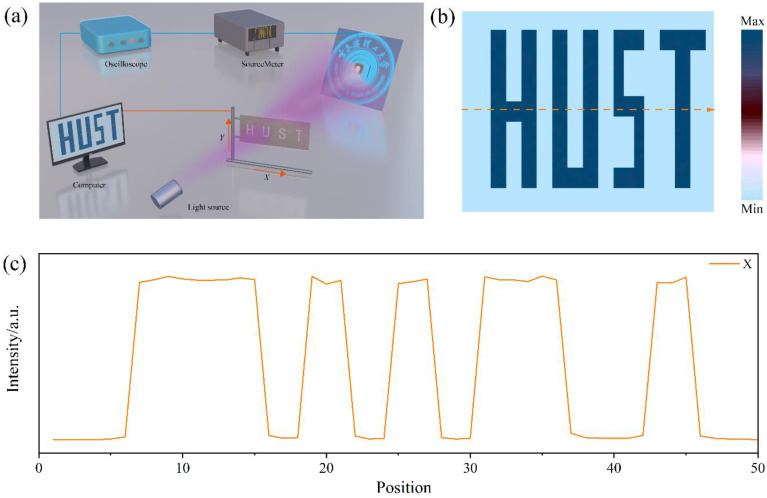
(**a**) Schematic illustration of the integrated imaging system. (**b**) Optical imaging results engraved with a “HUST” pattern and (**c**) the associated line scan current profile along the X-direction.

Capitalizing on the high-performance of the T-600 UVPD, it was incorporated as a sensing pixel in an imaging system to evaluate its optical imaging capabilities. As shown in Figure 8a, the T-600 device is positioned between the light source and a hollow “HUST” (the abbreviation of Harbin University of Science and Technology) mask, which automatically traverses along the X-Y direction. When the system is activated and exposed to UV light, the position-correlated *J* signal is immediately recorded via a source meter and oscilloscope, and the time-limited visualized image is displayed in real-time on a computer. As shown in Figure 8b, upon exposure to UV light (*λ* = 365 nm, *P_λ_* = 1 mW cm^−2^), a high-resolution “HUST” pattern image with clear boundaries was acquired. Furthermore, a linear scan of the *J* signal along the X-direction in the “HUST” imaging plot highlights the excellent dynamic optical extraction capability of the T-600 UVPD (Figure 8c).

**Figure 9:**
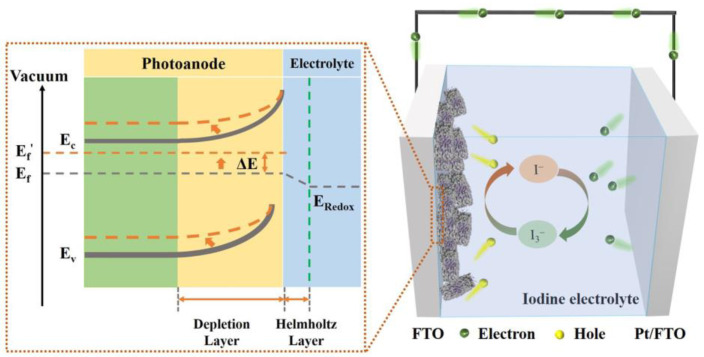
Schematic diagram of the photodetection mechanism for the T-600 PEC UVPD.

To assess the photo-induced change in the Fermi level (Δ*E*) for the three samples, measurements of open-circuit potential under both dark and light conditions were conducted (Figure S13). In the dark, a built-in electric field forms in the depletion layer due to the redistribution of inherent carriers in TiO_2_. Under illumination, photogenerated holes are consumed by absorbed anions in the Helmholtz layer, while electrons transfer to the interior, resulting in a decrease in the Fermi level. This process weakens the band bending under illumination. As shown in Figure 9, a larger Δ*E* implies a more efficient separation of photogenerated electron–hole pairs. The T-900 sample has a Δ*E* of 0.382 V, higher than that of the T-300 sample (0.301 V). Remarkably, the T-600 sample displays the highest Δ*E* value (0.562 V), indicating an optimal charge separation driving force in the depletion layer. The built-in electric field in T-600 expands the depletion layer, facilitating efficient charge separation. Based on the discussions above, a mechanism for the enhanced photodetection performance of the T-600 UVPD is proposed. When the T-600 sample absorbs UV light, the Type II band alignment of TiO_2_ A-R HHs and the presence of OVs facilitate the transfer of photogenerated carriers to the photoanode side, increasing the *J* of the device. At the same time, due to the existence of the barrier layer in the built-in electric field, the recombination loss of photogenerated carriers with I_3_^−^ in the Helmholtz layer can be effectively reduced, further improving the *J* of the device. Subsequently, the photogenerated electrons migrate along the external circuit toward the platinum-coated electrode, where they react with I_3_^−^ in the iodine electrolyte to form I^−^. The I^−^ diffusing in the electrolyte reacts with the holes on the sample surface to generate I_3_^−^. Ultimately, the continuous cycling of I^−^ and I_3_^−^ accomplishes a complete photoelectric detection process for the T-600 UVPD.

## 3. Materials and Methods

### 3.1. Synthesis of MIL-125(Ti)

MIL-125(Ti) was prepared by a solvothermal method according to the previously reported procedures [57]. In a typical experiment, terephthalic acid (TPA; 3.0 g) and absolute methanol (MeOH; 6 mL) were dissolved in N, N-dimethylmethanamide (DMF; 54.0 mL), and then stirred at ambient temperature for 1 h. Subsequently, titanium isopropoxide (TIP; 1.6 mL) was slowly added and constantly stirred for 30 min before being placed in an autoclave at 150 °C for 24 h. After cooling to room temperature, the product was rinsed with MeOH and DMF several times to remove all the unreacted organic ligand species. Eventually, the resulting powder was dried in air at 60 °C overnight to obtain MIL-125(Ti).

### 3.2. Synthesis of MOF-Derived TiO_2_

In this experiment, MIL-125(Ti) was used as a template, and its derivatives were obtained by calcining the precursor under different temperatures. For each sample, an alumina boat loaded with 2.0 g of as-prepared MIL-125(Ti) was positioned in a flow-through quartz tube furnace connected to Ar gas, with a flow rate set at 50 mL min^−1^. The heating ramp rate of the tube furnace was set at 5 °C min^−1^, and the system maintained a dwell time of 5 h at the target temperature. Subsequently, the samples were labeled T-x (x = 300, 600, and 900), where x represents the pyrolysis temperature.

### 3.3. Assembling of PEC UVPDs

Before fabricating the PEC UVPDs, an electrolyte was prepared following a procedure similar to our previous work [58]. A screen-printed film of MOF-derived TiO_2_, with an approximate area of 0.28 cm^2^ and a thickness of ~7.5 μm, served as the photoanode. A thermally platinized FTO electrode was employed as the counter electrode. The two electrodes were securely joined and sealed using a 30-μm-thick surlyn gasket under hot-pressing conditions. Finally, the injection hole was sealed hermetically using a mini hot press.

### 3.4. Characterizations

The morphology, elemental composition, and valence state of the samples were characterized using a field-emission scanning electron microscopy (FESEM; JEOL JEM-6700F), accompanied by an energy dispersive spectrometer (EDS) and X-ray photoelectron spectroscopy (XPS, Thermo Scientific K-Alpha). X-ray diffraction (XRD, Panalytical Empyrean) was applied to analyze the crystal structure. High-resolution transmission electron microscopy (HRTEM, JEOL JEM-2100) was utilized to investigate the microstructure of the samples. Raman spectra and UV–Vis absorption were recorded using a HORIBA HR Evolution and a Hitachi U-4100, respectively. Thermogravimetric (TG) analysis was conducted using an STA 409PC instrument. The specific surface area and pore size were characterized by N_2_ absorption–desorption measurements using an ASAP 2020 Micromeritics instrument. Electron paramagnetic resonances (EPR) were evaluated at room temperature using a Bruker EMX Micro spectrometer. Photocurrent density–voltage (J–V) and photocurrent response tests for the SPUs were performed using a Keithley 2602 SourceMeter. Monochromatic light was generated by splitting a 500 W xenon light source using an automatic grating monochromator. A standard monocrystalline silicon solar cell was employed as a reference for calibration and accuracy checks. Photoluminescence (PL) and time-resolved photoluminescence (trPL) spectra were acquired using an Edinburgh FLS1000. Electrochemical impedance spectroscopy (EIS) and Mott–Schottky (M–S) analysis were conducted on a CHI660E electrochemical workstation.

## 4. Conclusions

In summary, we have synthesized TiO_2_ A-R HHs featuring OVs via an innovative in situ pyrolysis approach employing Ti-MOFs and have applied them as photoanodes in PEC UVPDs. Our experimental data reveal that the built-in electric field within TiO_2_ A-R HHs not only ensures effective photogenerated carrier separation, but also reduces the recombination loss of photogenerated carriers. Moreover, the introduction of OVs serves as electron donors, increasing the electric conductivity and thus promoting the separation and transfer of photogenerated carriers. Taking advantage of the combined benefits of improved carrier separation and reduced carrier recombination, the TiO_2_ PEC UVPD exhibits a high on/off ratio of 10,752, a remarkable responsivity of 24.15 mA W^−1^, an impressive detectivity of 3.28 × 10^11^ Jones, and excellent cycling stability. Notably, under UV light irradiation, this device successfully captured a clear two-dimensional image with a “HUST” pattern, confirming its outstanding optical imaging capability. Given these remarkable photodetection properties, it is logical to posit that the UVPDs engineered from MOF-derived TiO_2_ A-R HHs exhibit considerable potential for high-performance, multi-purpose optoelectronic systems.

## Figures and Tables

**Table 1:** Pore textural, phase contents, and composition properties of the as-prepared samples.

Sample	Surface Area (m^2^ g^−1^)	Pore Size (nm)	W_A_/W_R_ (%)	A-Phase Size (nm)	R-Phase Size (nm)	Carbon Content (%)
MIL-125(Ti)	921.1	3.2	^−^	^−^	^−^	43.8
T-300	246.7	3.6	^−^	10.5	^−^	38.5
T-600	23.7	20.5	49.9/50.1	19.3	21.1	13.6
T-900	1.4	17.4	0	^−^	66.2	8.7

## Data Availability

The original contributions presented in the study are included in the article/Supplementary Materials, further inquiries can be directed to the corresponding authors.

## Supplementary Materials

The following supporting information can be downloaded at: https://www.mdpi.com/article/10.3390/molecules29133096/s1, Figure S1: XRD patterns of the simulated and as-synthesized MIL-125(Ti); Figure S2: Nitrogen adsorption–desorption isotherms (a–d) and pore size distribution plot (e–h) of MIL-125(Ti), T-300, T-600, and T-900; Figure S3: SEM image of MIL-125(Ti); Figure S4: EDS analysis of (a) MIL-125(Ti), (b) T-300, (c) T-600, and (d) T-900 respectively; Figure S5: Magnification of the Raman patterns in the 100–200 cm^−1^ for the as-prepared samples; Figure S6: XPS survey spectra of T-300, T-600, and T-900; Figure S7: M–S plots of T-300, T-600, and T-900; Figure S8: Enlarged rising and decaying edges of the photocurrent response for the PEC UVPDs with (a) T-300 and (b) T-900; Figure S9: Photocurrent response of T-600 PEC UVPD as a function of time under UV light intensities from 5.03 to 45.61 mW cm^−2^; Figure S10: The trPL spectra of T-600 irradiated by 254 and 365 nm; Figure S11: Spectral responsivity characteristic for the PEC UVPDs with T-300, T-600 and T-900; Figure S12: (a) *J*−*V* characteristics and (b) thermal stability measurement of the PEC UVPD with T-600 (*J*_sc_ and *V*_oc_ as a function of time); Figure S13: Open-circuit potential of (a) T-300, (b) T-500, and (c) T-900 photoanodes in Na_2_SO_4_ with light off and light on, respectively; Table S1: The quantitative analysis results of the C chemical bonds and the peak area ratio (O_II_/O_I_+O_II_) obtained through XPS for the as-prepared samples; Table S2: Comparison of nanomaterial-based PEC UVPDs.
