# Supplementary material for: Optimizing Photoelectrochemical UV Imaging Photodetection: Construction of Anatase/Rutile Heterophase Homojunctions and Oxygen Vacancies Engineering in MOF-Derived TiO2
Source: Molecules. 2024 Jun 28;29(13):3096. doi: 10.3390/molecules29133096 (PMC11243629; doi:10.3390/molecules29133096)
Supplement: Supplementary file 1 [file molecules-29-03096-s001.zip › molecules-3061054-supplementary.pdf]

Optimizing Photoelectrochemical UV Imaging Photodetection: Construction of Anatase/Rutile Heterophase Homojunctions and Oxygen Vacancies Engineering in MOF-Derived TiO_2

Yueying Ma[1], Yuewu Huang[2,*], Ju Huang[1], Zewu Xu[2], Yanbin Yang[2], Changmiao Xie[2], Bingke Zhang[3], Guanghong Ao[1,*], Zhendong Fu[4], Aimin Li[4], Dongbo Wang[3,*] and Liancheng Zhao[3]

[1]*School of Science, Harbin University of Science and Technology, Harbin 150080, China.*

[2]*School of Materials Science and Chemical Engineering, Harbin University of Science and Technology, Harbin 150080, China.*

[3]*Department of Opto-electronic Information Science, School of Materials Science and Engineering, Harbin Institute of Technology, Harbin 150080, China.*

[4]*Tianjin Jinhang Technical Physics Institute, Tianjin 300308, China*

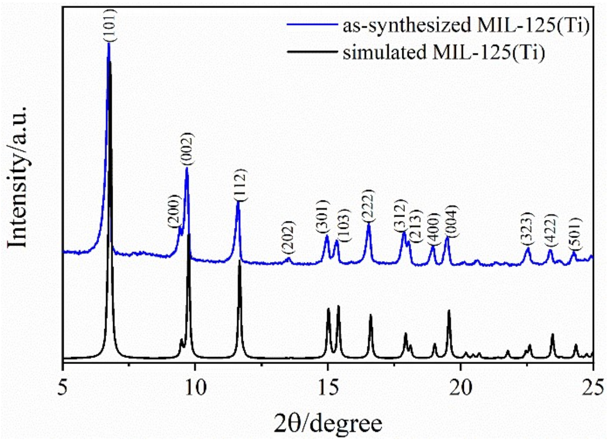

Figure S1. XRD patterns of the simulated and as-synthesized MIL-125(Ti).

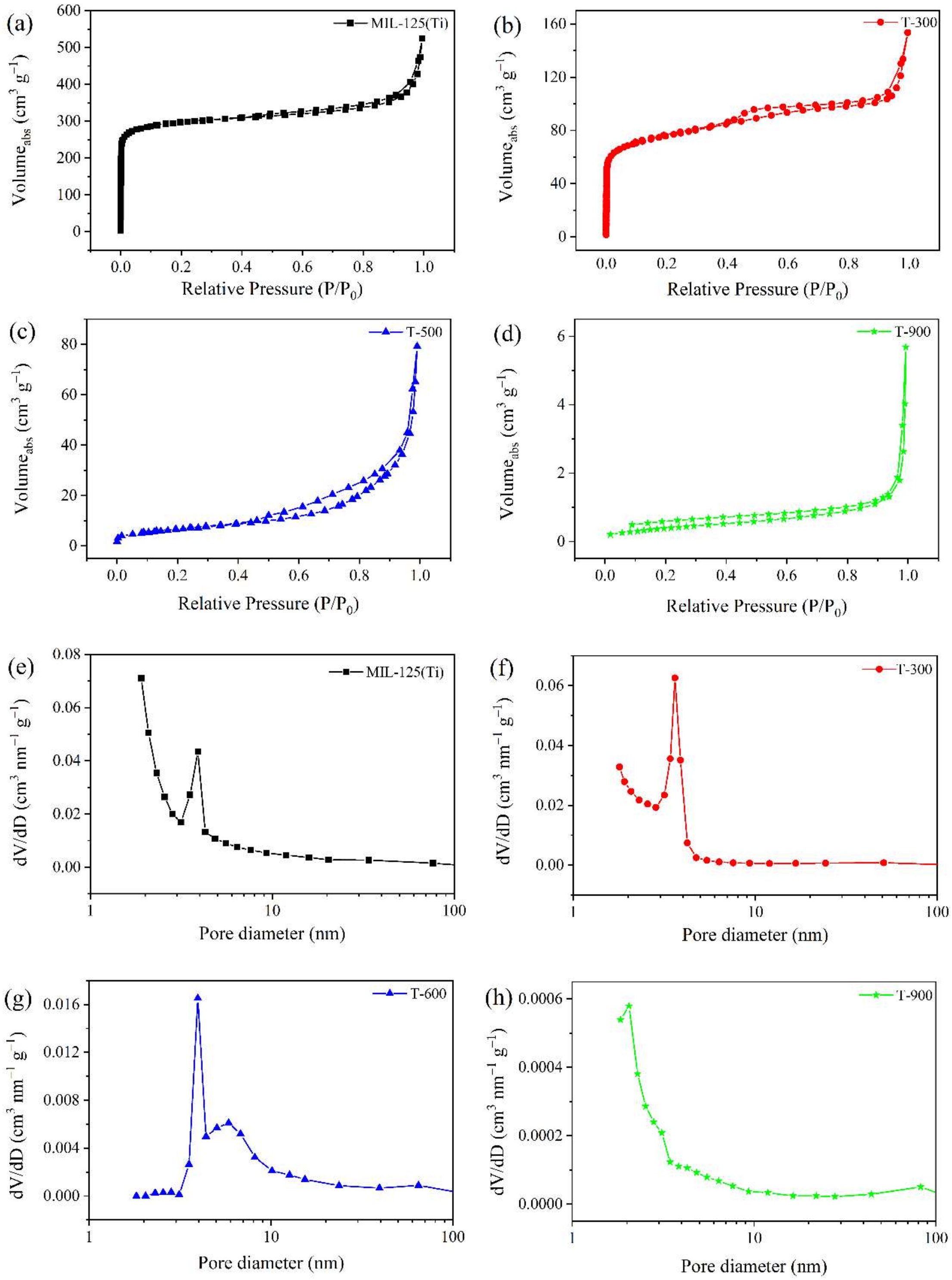

Figure S2. Nitrogen adsorption–desorption isotherms (a-d) and pore size distribution plot (e-h) of MIL-125(Ti), T-300, T-600, and T-900.

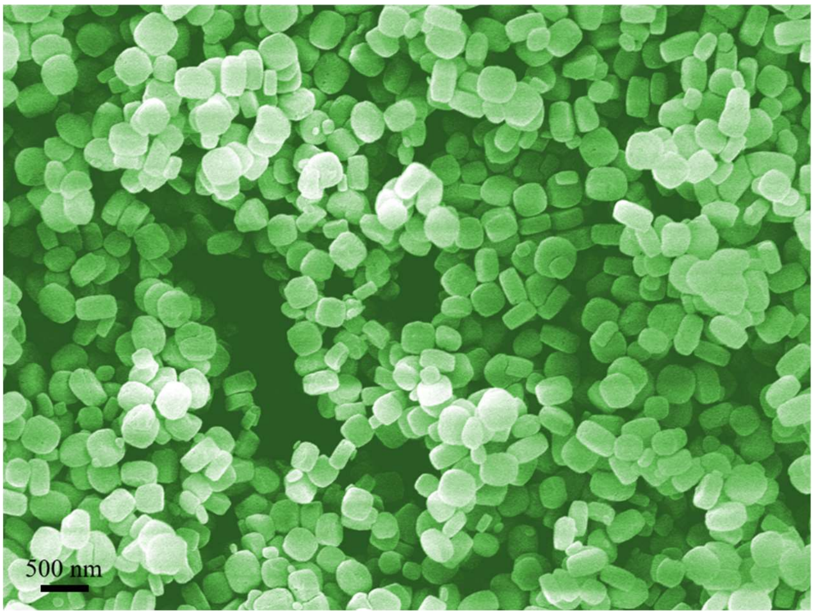

Figure S3. SEM image of MIL-125(Ti).

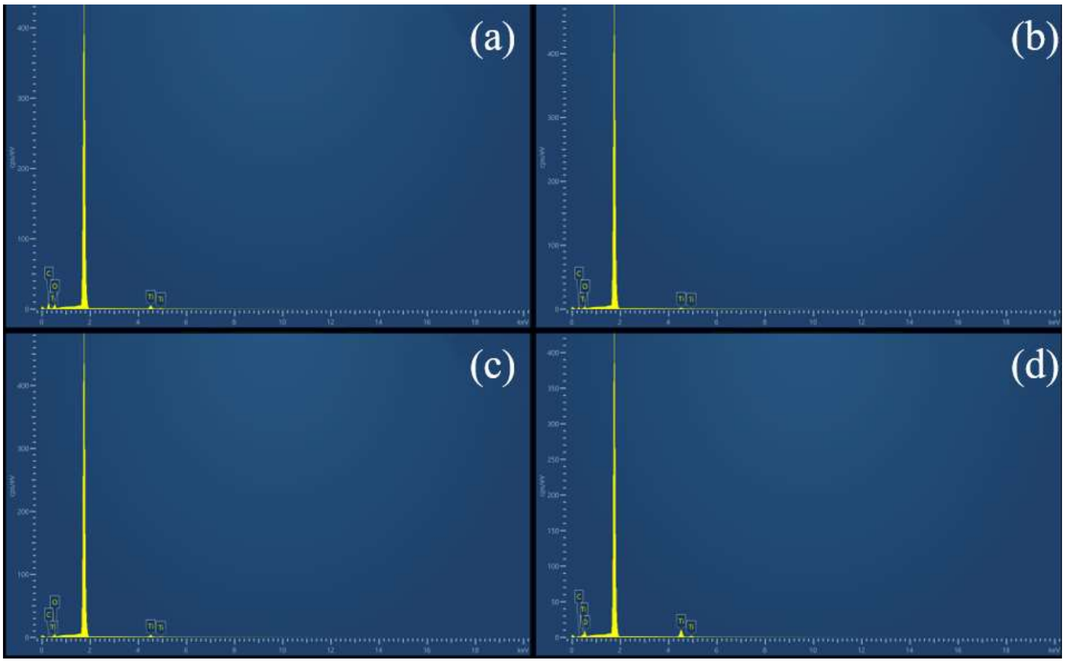

Figure S4. EDS analysis of (a) MIL-125(Ti), (b) T-300, (c) T-600, and (d) T-900 respectively.

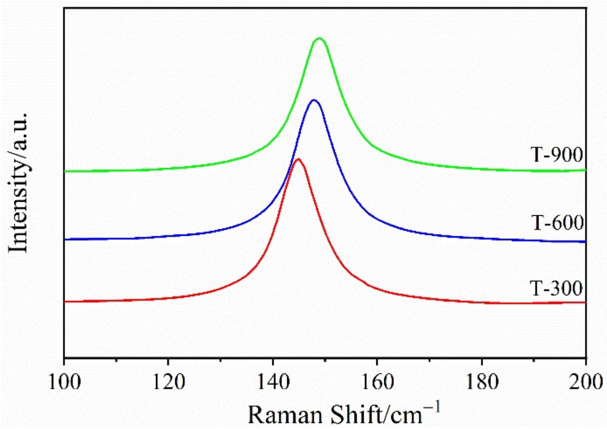

Figure S5. Magnification of the Raman patterns in the 100-200 cm^{-1} for the as-prepared samples.

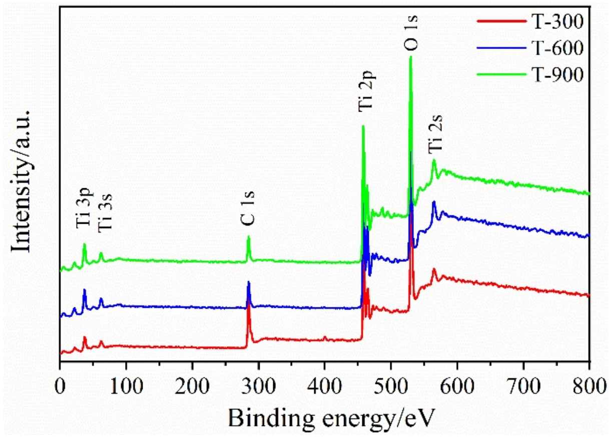

Figure S6. XPS survey spectra of T-300, T-600, and T-900.

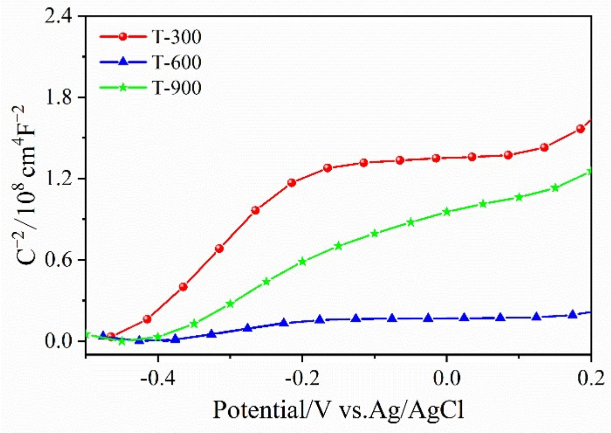

Figure S7. M–S plots of T-300, T-600, and T-900.

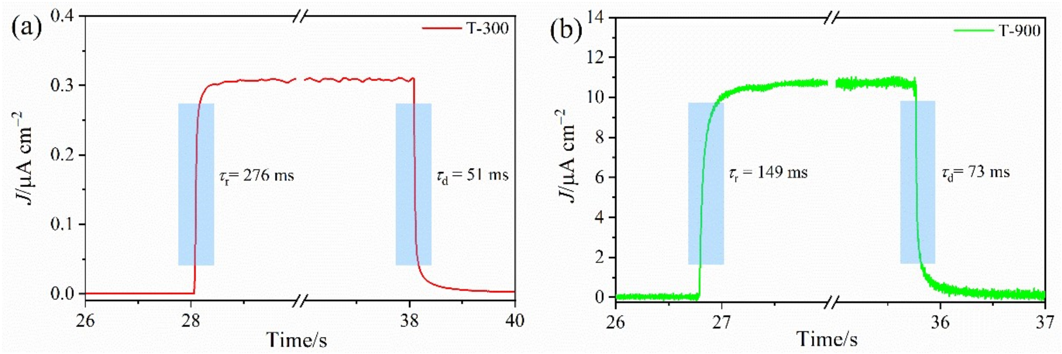

Figure S8. Enlarged rising and decaying edges of the photocurrent response for the PEC UVPDs with (a) T-300 and (b) T-900.

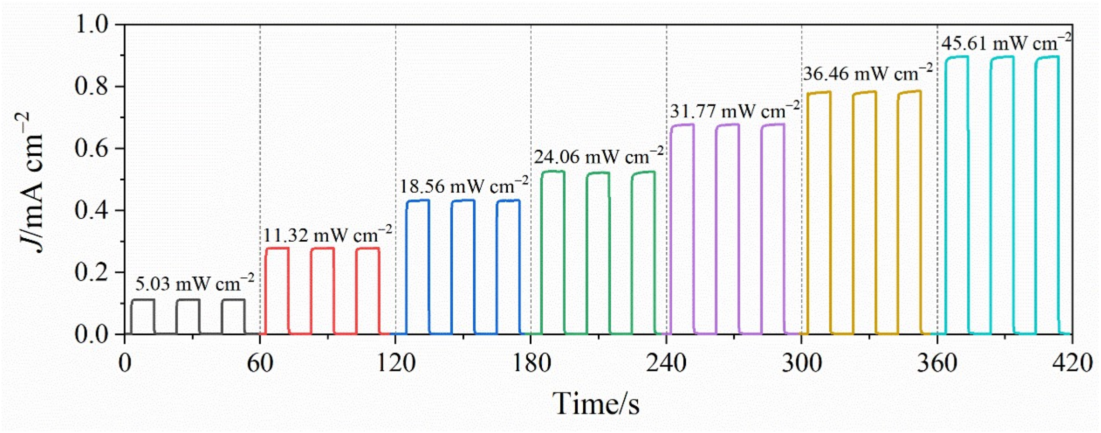

Figure S9. Photocurrent response of T-600 PEC UVPD as a function of time under UV light intensities from 5.03 to 45.61 mW cm^{-2}.

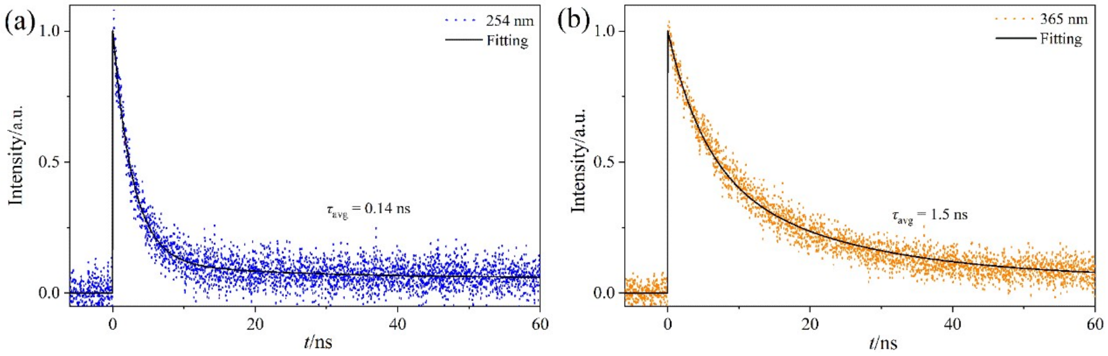

Figure S10. The trPL spectra of T-600 irradiated by 254 and 365 nm.

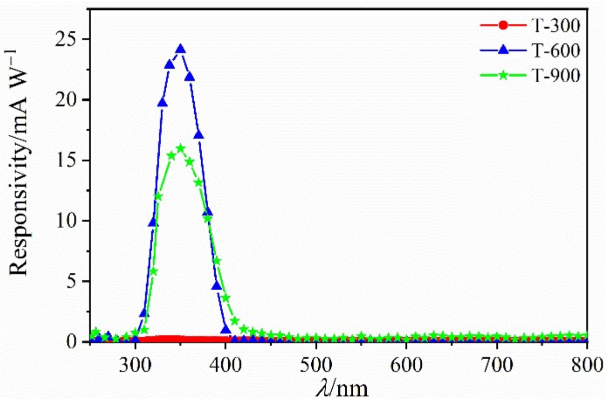

Figure S11. Spectral responsivity characteristic for the PEC UVPDs with T-300, T-600 and T-900.

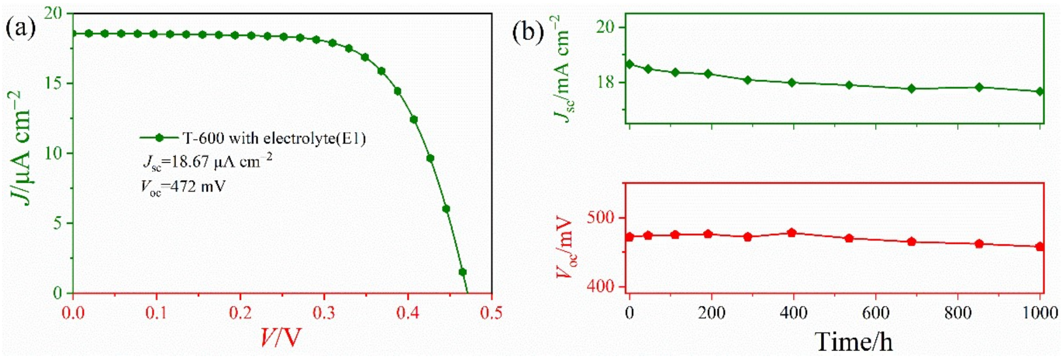

Figure S12. (a) *J*–*V* characteristics and (b) thermal stability measurement of the PEC UVPD with T-600 (J_{sc} and V_{oc} as a function of time).

*Electrolytes (E1): 1.0 M DMII, 0.15 M I_2, 1.0 M TBP, 50 mM LiI, and 0.1 M GNCS in 3-methoxypropinitrile.

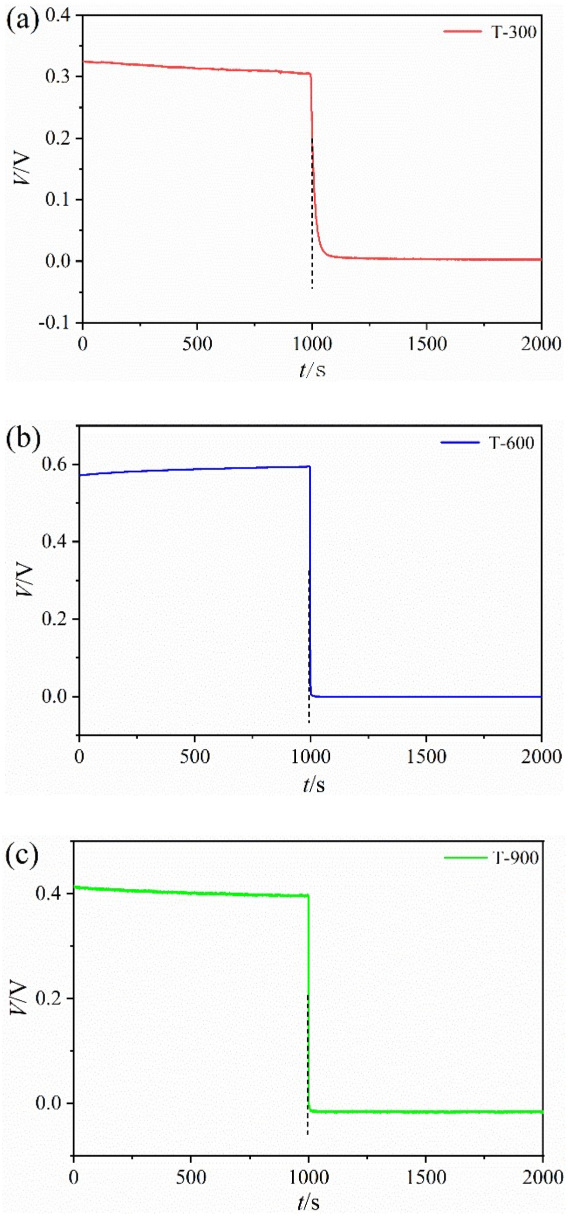

Figure S13. Open-circuit potential of (a) T-300, (b) T-500, and (c) T-900 photoanodes in Na_2SO_4 with light off and light on, respectively.

Table S1. The quantitative analysis results of the C chemical bonds and the peak area ratio (O_{II}/O_I+O_{II}) obtained through XPS for the as-prepared samples.

Sample	C-C (%)	C-O (%)	C=O (%)	$O_{II}/(O_I+O_{II})$ (%)
T-300	67.9	23.5	9.2	16.2
T-600	69.3	22.3	7.8	9.9
T-900	72.1	21.1	6.8	11.4

Table S2. Comparison of nanomaterial-based UVPDs.

Materials	Measurement condition	Light (nm)	Rise/decay time (ms)	R_λ (mA W^{-1})	D^* (Jones)	Ref
$Ti_3C_2T_x/TiO_2$	1 M, LiI, 0 V	360	45/69	2.06	-	S1
ZnO NNs	Water, 0 V	375	100/100	22	-	S2
TiO_2 NAs	I^-/I_3^-, 0 V	365	13/19	0.075	-	S3
α-Ga_2O_3 NAs	0.1 M NaOH, 0 V	245	76/56	0.21	-	S4
TiO_2 NAs	Water, 0 V	365	150/50	25	-	S5
$SrTiO_3$-BL	I^-/I_3^-, 0 V	365	6/8	2.8	-	S6
TiO_2@Al_2O_3	0.5 M Na_2SO_4, 0 V	365	90/90	2.4	-	S7
ZnO/G/Cu_2O	0.5 M Na_2SO_4, 0 V	365	6/6	21.2	-	S8
Ga_2O_3/Al_2O	0.1 M NaOH, 0 V	245	<100/<100	0.174	-	S9
2D boron	0.3 M KOH, 0.2 V	365	100/200	0.0675	0.97×10^8	S10
ZnO/CuSCN/rGO	17.7 mW cm^{-2}, −1 μV	375	105/100	18.65	3.7×10^{11}	S11
TiO_2/MoO_3	0.076 mW cm^{-2}, 0 V	352	1820/1480	108	2.26×10^{10}	S12
PbS	0.1 M KOH, 0.4 V	400	160/150	10.97	3.96×10^{10}	S13
MOF-In_2O_3	0.01M Na_2SO_4, 0.6 V	365	500/1100	21.9	2.03×10^{10}	S14
TiO_2-$BaTiO_3$ NRs	2 mW cm^{-2}, 0 V	375	2.1/1.4	3.34	7.96×10^{10}	S15
Bi_2O_3/TiO_2	2 mW cm^{-2}, 0 V	405	7/65	1.79	5.94×10^{10}	S16
Bi_2O_3/Ag/TiO_2	I^-/I_3^-, 0 V	365	27/28	1.72	1.55×10^{10}	S17
TiO_2 NRs/Au/PTTh	0.41 mW cm^{-2}, 0 V	365	230/280	1.894	1.666×10^{10}	S18
p-CuI/n-TiO_2	1.61 mW cm^{-2}, 5 V	310	0.11/0.72	0.6	8.4×10^{11}	S19
Y^{3+}-doped TiO_2	0.62 W cm^{-2}, 3 V	350	2.53/1.16	4500	1.6×10^{11}	S20
TiO_2/NiO	1.2 W cm^{-2}, 0 V	365	1200/7100	0.042	1.1×10^9	S21
SnS_x/TiO_2	2 mW cm^{-2}, 0 V	365	3/25-46	5.9	6.5×10^{10}	S22
TiO_2/P3HT	–, 0 V	350	720/500	0.037	1.63×10^{10}	S23
ZnO/Spiro-MeOTAD	1 mW cm^{-2}, 0 V	365	160/200	0.8	4.2×10^9	S24
Vo-Ga_2O_3/ZnO	0.5 M Na_2SO_4, 0 V	266	150/1100	7.975	1.16×10^{11}	S25
GaN NAs	0.5 M H_2SO_4, 0 V	365	145/81	6.04	5.4×10^{10}	S26
Bi QDs	0.1 M KOH, 0 V	365	100/200	0.0193	9.09×10^8	S27
Te	0.1 M KOH, 0.6 V	350	54.5/70.2	0.0134	3.1×10^7	S28
Bi_2S_3	0.1 M KOH, 0.6 V	400	100/100	0.011	3.75×10^8	S29
MOF-TiO_2 A-R HHs	I^-/I_3^-/ 0 V	6.0 μA	50/108	24.15	3.28×10^{11}	This wok

NNAs: nanoneedls; NTs: nanotubes; NWs: nanostructures; NSs: nanosheets; NC:nanocrystalline; QDs: quantum dots; NPLs: nanoplatelets; BL:biock layer; NRs: nanorod arrays

References

[S1] Ma, H.L.; Jia, L.; Lin, Y.A.; Fang, H.J.; Wu, W.T.; Wu, L.L.; Hu, B.; Wang, H. A self-powered photoelectrochemical ultraviolet photodetector based on $Ti_3C_2T_x/TiO_2$ in situ formed heterojunctions. *Nanotechnology* **2022**, *33*, 075502. https://doi.org/10.1088/1361-6528/ac0eaa.

[S2] Li, Q.H.; Wei, L.; Xie, Y.R.; Zhang, K.; Liu, L.; Zhu, D.P.; Jiao, J.; Chen, Y.X.; Yan, S.S.; Liu, G.L.; Mei, L.M. ZnO nanoneedle/H_2O solid-liquid heterojunction-based self-powered ultraviolet detector. *Nanoscale Res.* Lett. **2013**, *8*, 415. https://doi.org/10.1186/1556-276X-8-415.

[S3] Wang, G.; Bai, Z.W.; Wu, H.C.; Zhang, X.M.; Li, J.; Jin, M.J.; Zhou, J.Y.; Xie, E.Q.; Pan, X.J. A wire-shaped and high-sensitivity photoelectrochemical ultraviolet photodetector based on TiO_2 nanotube arrays. *Appl. Phys. Lett.* **2022**, *121*, 11101. https://doi.org/10.1063/5.0102834.

[S4] Zhang, J.H.; Jiao, S.J.; Wang, D.B.; Ni, S.M.; Gao, S.Y.; Wang, J.Z. Solar-blind ultraviolet photodetection of an α-Ga_2O_3 nanorod array based on photoelectrochemical self-powered detectors with a simple, newly-designed structure. *J. Mater. Chem.* C **2019**, *7*, 6867–6871. https://doi.org/10.1039/c9tc01417a.

[S5] Xie, Y.R.; Wei, L.; Wei, G.D.; Li, Q.H.; Wang, D.; Chen, Y.X.; Yan, S.S.; Liu, G.L.; Mei, L.M.; Jiao, J. A self-powered UV photodetector based on TiO_2 nanorod arrays. *Nanoscale Res. Lett.* **2013**, *8*, 188. https://doi.org/10.1186/1556-276X-8-188.

[S6] Zhu, Z.R.; Wei, K.; Li, H.; Li, X.P.; Li, B.P.; Gu, X.Y.; Chen, L.L.; Zhou, J.Y.; Pan, X.-J.; Wang, Y.-Q. High-sensitivity photoelectrochemical visible-blind ultraviolet detector using $SrTiO_3$ nanocrystalline for weak irradiation. *J. Phys. D-Appl. Phys.* **2021**, *54*, 095104. https://doi.org/10.1088/1361-6463/abc8b6.

[S7] Wang, G.; Ji, F.B.; Li, J.; Z, X.M.; Wu, H.C.; Bai, Z.W.; Jin, M.J.; Zhou, J.Y.; Xie, E.Q. Preparation of a flexible photoanode of the photoelectrochemical ultraviolet photodetector based on rutile TiO_2 nanowires and the suppressed charge recombination at solid/liquid interface. *J. Phys.* D **2024**, *57*, 025101. https://doi.org/10.1088/1361-6463/acffd6.

[S8] Bai, Z.M.; Liu, J.; Liu, F.X.; Zhang, Y.H. Enhanced photoresponse performance of self-powered UV–visible photodetectors based on ZnO/Cu_2O/electrolyte heterojunctions via graphene incorporation. *J. Alloy. Compd.* **2017**, *726*, 803–809. https://doi.org/10.1016/j.jallcom.2017.08.035.

[S9] Zhang, J.H.; Jiao, S.J.; Wang, D.B.; Gao, S.Y.; Wang, J.Z.; Zhao, L.C. Nano tree-like branched structure with α-Ga_2O_3 covered by γ-Al_2O_3 for highly efficient detection of solar-blind ultraviolet light using self-powered photoelectrochemical method. *Appl. Surf. Sci.* **2021**, *541*, 148380. https://doi.org/10.1016/j.apsusc.2020.148380.

[S10] Ma, D.T.; Wang, R.; Zhao, J.L.; Chen, Q.Y.; Wu, L.M.; Li, D.L.; Su, L.M.; Jiang, X.T.; Luo, Z.Q.; Ge, Y.Q.; Li, J.Q.; Zhang, Y.P.; Zhang, H. A self-powered photodetector based on two-dimensional boron nanosheets. *Nanoscale* **2020**,

12, 5313–5323. https://doi.org/10.1039/d0nr00005a.
[S11]Luo, G.C.; Zhang, Z.L.; Jiang, J.; Liu, Y.; Li, W.; Zhang, J.Q.; Hao, X.; Wang, W.W. Enhanced performance of ZnO nanorod array/CuSCN ultraviolet photodetectors with functionalized graphene layers. *RSC Adv.* **2021**, *11*, 7682–7692. https://doi.org/10.1039/d0ra10420e.
[S12]Ezhilmaran, B.; Dhanasekar, M.; Bhat, S.V. Solution processed transparent anatase TiO_2 nanoparticles/MoO_3 nanostructures heterojunction: high performance self-powered UV detector for low-power and low-light applications. *Nanoscale Adv.* **2021,** *3,* 1047–1056. https://doi.org/10.1039/d0na00780c.
[S13]Gao, L.F.; Chen, H.L.; Wang, R.; Wei, S.R.; Kuklin, A.V.; Mei, S.; Zhang, F.; Zhang, Y.; Jiang, X.T.; Luo, Z.Q.; Xu, S.X.; Zhang, H.; Ågren, H. Ultra-Small 2D PbS Nanoplatelets: Liquid-Phase Exfoliation and Emerging Applications for Photo-Electrochemical Photodetectors. *Small* **2021,** *17* 2005913. https://doi.org/10.1002/smll.202005913.
[S14]Cui, M.Q.; Shao, Z.T.; Qu, L.H.; Liu, X.; Yu, H.; Wang, Y.X.; Zhang, Y.X.; Fu, Z.D.; Huang, Y.W.; Feng, W. MOF-Derived In_2O_3 Microrods for High-Performance Photoelectrochemical Ultraviolet Photodetectors. *ACS Appl. Mater. Interfaces* **2022**, *14*, 39046–39052. https://doi.org/10.1021/acsami.2c09968.
[S15]Wang, Z.; Xu, J.P.; Shi, S.B.; Chen, J.; Xu, J.H.; Kong, L.A.; Zhang, X.S.; Li, L.; Yin, S.G. Self-Powered UV Photodetector of TiO_2 with $BaTiO_3$ Surface Modification and Light-Controlled Logic Circuits Application. *ACS Appl. Mater. Interfaces* **2023**, *15*, 31943–31953. https://doi.org/10.1021/acsami.3c03628.
[S16]Zheng, Q.; Xu, J.P.; Shi, S.B.; Chen, J.; Xu, J.H.; Kong, L.N.; Zhang, X.S.; Li, L. Improved performance of UV-blue dual-band Bi_2O_3/TiO_2 photodetectors and application of visible light communication with UV light encryption. *Phys. Chem. Chem. Phys.* **2023**, *25*, 30228–30236. https://doi.org/10.1039/d3cp04100j.
[S17]Ren, S.; Gao, S.Y.; Lu, H.Q.; Rong, P.; Li, L.; Zhang, M.Y.; Xu, B.T.; He, W.; Jiao, S.J.; Wang, J.Z. Interface engineering of Bi_2O_3/Ag/TiO_2 nanotubes heterojunction photodetector for UV imaging and optical communication. *Appl. Surf. Sci.* **2024**, *643*, 158649. https://doi.org/10.1016/j.apsusc.2023.158649.
[S18]Zhang, H.J.; Abdiryim, T.; Jamal, R.; Li, J.X.; Liu, H.-L.; Kadir, A.; Zou, D.-N.; Che, Y.Z.; Serkjan, N. Self-powered TiO_2 NRs UV photodetectors: Heterojunction with PTTh and enhanced responsivity by Au nanoparticles. J. Alloy. Compd. **2022**, *899*, 163279. https://doi.org/10.1016/j.jallcom.2021.163279.
[S19]Zuo, C.L.; Cai, S.; Li, Z.L.; Fang, X.S. A transparent, self-powered photodetector based on p-CuI/n-TiO_2 heterojunction film with high on–off ratio. *Nanotechnology* **2022**, *33*, 105202. https://doi.org/10.1088/1361-6528/ac3e35.
[S20]Li, Z.L.; Joshi, M.K.; Chen, J.X., Zhang, Z.M.; Li, Z.Q.; Fang, X.S. Mechanically Compatible UV Photodetectors Based on Electrospun Free-

Standing Y^{3+}-Doped TiO_2 Nanofibrous Membranes with Enhanced Flexibility. *Adv. Funct. Mater.* **2020**, *30*, 2005291. https://doi.org/10.1002/adfm.202005291.

[S21]Zheng, L.X.; Teng, F.; Zhang, Z.M.; Zhao, B.; Fang, X.S. Large scale, highly efficient and self-powered UV photodetectors enabled by all-solid-state n-TiO_2 nanowell/p-NiO mesoporous nanosheet heterojunctions. *J. Mater. Chem.* C **2016**, *4*, 10032–10039. https://doi.org/10.1039/c6tc03830a.

[S22]Chen, J.; Xu, J.P.; Shi, S.B.; Cao, R.; Liu, D.; Bu, Y.C.; Yang, P.C.; Xu, J.H.; Zhang, X.S.; Li, L. Novel Self-Powered Photodetector with Binary Photoswitching Based on SnS_x/TiO_2 Heterojunctions. **ACS Appl. Mater. Interfaces 2020**, *12*, 23145–23154. https://doi.org/10.1021/acsami.0c05247.

[S23]Zheng, L.X.; Deng, X.L.; Wang, Y.Z.; Chen, J.X.; Fang, X.S.; Wang, L.; Shi, X.W.; Zheng, H.J. Self-Powered Flexible TiO_2 Fibrous Photodetectors: Heterojunction with P3HT and Boosted Responsivity and Selectivity by Au Nanoparticles. *Adv. Funct. Mater.* **2020**, *30*, 2001604. https://doi.org/10.1002/adfm.202001604.

[S24]Shen, Y.W.; Yan, X.Q.; Si, H.N.; Lin, P.; Liu, Y.C.; Sun, Y.H.; Zhang, Y. Improved Photoresponse Performance of Self-Powered ZnO/Spiro-MeOTAD Heterojunction Ultraviolet Photodetector by Piezo-Phototronic Effect. *ACS Appl. Mater. Interfaces* **2016**, *8*, 6137–6143. https://doi.org/10.1021/acsami.5b12870.

[S25]Ni, D.W.; Wang, Y.J.; Li, A.S.; Huang, L.; Tang, H.L.; Liu, B.; Cheng, C.W. ALD oxygen vacancy-rich amorphous Ga_2O_3 on three-dimensional urchin-like ZnO arrays for high-performance self-powered solar-blind photodetectors. *Nanoscale* **2022**, *14*, 3159–3165. https://doi.org/10.1039/d1nr08262k.

[S26]Chen, K.; Zhang, D.Q.; Shao, P.F.; Zhi, T.; Zhao, J.G.; Sang, Y.M.; Hu, W.X.; Ye, Y.C.; Xue, J.J.; Chen, D.J.; Tao, T.; Liu, B. Ordered GaN Nanorod Arrays for Self-Powered Photoelectrochemical Ultraviolet Photodetectors. *ACS Appl. Nano Mater.* **2022**, *5*, 13149–13157. https://doi.org/10.1021/acsanm.2c02910.

[S27]Xing, C.Y.; Huang, W.C.; Xie, Z.J.; Zhao, J.L.; Ma, D.T.; Fan, T.J.; Liang, W.Y.; Ge, Y.Q.; Dong, B.Q.; Li, J.Q.; Zhang, H. Ultrasmall Bismuth Quantum Dots: Facile Liquid-Phase Exfoliation, Characterization, and Application in High-Performance UV–Vis Photodetector. *ACS Photonics* **2018**, *5*, 621–29.https://doi.org/10.1021/acsphotonics.7b01211.

[S28]Xie, Z.J.; Xing, C.Y.; Huang, W.C.; Fan, T.J.; Li, Z.J.; Zhao, J.L.; Xiang, Y.J.; Guo, Z.N.; Li, J.Q.; Yang, Z.G.; Dong, B.Q.; Qu, J.L.; Fan, D.Y.; Zhang, H. Ultrathin 2D Nonlayered Tellurium Nanosheets: Facile Liquid-Phase Exfoliation, Characterization, and Photoresponse with High Performance and Enhanced Stability. *Adv. Funct. Mater.* **2018**, *28*, 170583. https://doi.org/10.1002/adfm.201705833.

[S29]Huang, W.C.; Xing, C.Y.; Wang, Y.Z.; Li, Z.J.; Wu, L.M.; Ma, D.T.; Dai, X.Y.; Xiang, Y.J.; Li, J.Q.; Fan, D.Y.; Zhang, H. Facile fabrication and characterization of two-dimensional bismuth (III) sulfide nanosheets for high-performance photodetector applications under ambient conditions.

Nanoscale **2018**, *10*, 2404–2412. https://doi.org/10.1039/c7nr09046c.
